# Synchronous presentation of COVID‐19 pneumonia and pulmonary embolism

**DOI:** 10.1002/ccr3.3870

**Published:** 2021-01-27

**Authors:** Farid Poursadegh, Najmeh Davoudian, Mahnaz Mozdourian, Fahimeh Abdollahi

**Affiliations:** ^1^ Lung diseases Research Center Mashhad University of Medical Sciences Mashhad Iran; ^2^ Department of Internal Medicine Clinical Research Development Unit Faculty of Medicine Bohlool Hospital Gonabad University of Medical Sciences Gonabad Iran

**Keywords:** COVID‐19, hypercoagulable states, pneumonia, Pulmonary embolism

## Abstract

The patients with COVID‐19 pneumonia who suffer from worsening of the clinical respiratory symptoms, after the beginning of the treatment, should be evaluated for pulmonary embolism using CT angiography if there are no contraindications.

## Graphical abstract

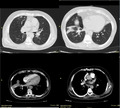

## 1 INTRODUCTION

Simultaneous diagnosis of COVID‐19 pneumonia and pulmonary embolism without any deep vein thrombosis nor predisposing hypercoagulable states was observed. Therefore, patients with COVID‐19 pneumonia who suffer from worsening of the clinical respiratory symptoms, after the beginning of the treatment, should be evaluated for pulmonary embolism using CT angiography, if safe.

In late 2019, a novel coronavirus named COVID‐19 led to a large outbreak in China and many other countries.^1^, ^2^ Severe acute respiratory syndrome coronavirus 2 (SARS‐CoV‐2; formerly called 2019‐nCoV) is a major health concern that is mainly presented by the involvement of respiratory and gastrointestinal systems.^3^ There are reports on potential increased risks of thrombotic events such as pulmonary embolism and deep vein thrombosis, in critically ill intensive care unit (ICU) patients with COVID‐19.^4^, ^5^ However, the presentation of these thrombotic complications at admission has been reported, rarely.^6^, ^7^ Pulmonary thromboembolism is caused by the development of a clot in the circulatory system and its subsequent deposition on the branches of the pulmonary arteries. Mortality due to pulmonary embolism has been reported annually and is therefore considered as one of the most important life‐threatening factors.^8^

Acute pulmonary thromboembolism is a common pulmonary and cardiac disorder that may arise from a mild and even asymptomatic life‐threatening illness. However, acute pulmonary thromboembolism is a fatal disease. The signs and symptoms of thromboembolism are all nonspecific. It also mimics the symptoms of many diseases. Deep thrombophlebitis, immobility, long journeys, obesity, birth control pills, etc, are predisposing factors for pulmonary embolism. Global inflammation in the body, hospitalization, and immobility and increased coagulation factors are additional predisposing factors for thrombosis and pulmonary embolism in COVID‐19 patients.^9^

We report a middle‐aged man presenting with the synchronous diagnosis of COVID‐19 pneumonia and pulmonary embolism in the absence of any deep vein thrombosis nor predisposing hypercoagulable states.

## 2 CASE PRESENTATION

A 72‐year‐old man with a past medical history of well‐controlled diabetes mellitus and hypertension presented to the emergency department with a history of fatigue, muscle weakness, sore throat, fever, and chills for the last two weeks. The severity of symptoms has exacerbated recently and suffered from shortness of breath. At the presentation, he was lethargic and suffering tachycardia (heart rate of 108 beats/min), and tachypneic (respiratory rate of 30 breaths/min) with a temperature of 38.4°C and blood pressure of 190/60 mm Hg. Also, he had a low oxygen saturation level (SpO2 of 85%). Physical examination of the right lung using auscultation revealed fine crackles. The laboratory test results showed lymphopenia (624 cellsper cubic millimeter of blood) and C‐reactive protein (CRP) level of 79 mg/dL. The liver and kidney function tests and serum electrolyte panel were all normal. The concentrations of biochemical parameters were 231 µg/ml creatine phosphokinase (CPK‐MB), 1.3 µg/ml troponin I, and 468 units per liter (U/L) lactic acid dehydrogenase (LDH) (Table 1).

**TABLE 1:** The laboratory test results of the patient at the time of admission

Measure	Result
WBC (×10⁹/L)	3.9
Neut (×10⁹/L)	75
Lym (×10⁹/L)	16
Hb (g/dL)	17
Plt (×10⁹/L)	222
ESR (mm/h)	45
CRP (mg/dL)	79
BUN (mg/dL)	76
Cr (mg/dL)	1.4
Na (mEq/L)	136
K (mEq/L)	3.9
CPK (mg/dL)	231
AST (units/L)	49
ALT (units/L)	57
ALP (units/L	198
Total bilirubin (μmol/L)	1.1
Direct bilirubin (μmol/L)	0.17
D‐dimer (ng/mL)	1120
LDH (units/L)	468
PT (seconds)	12.8
PTT (seconds)	37
INR	1.09
VBG (primary)	
PH	7.40
PCO2 (mm Hg)	47
HCO3 (mEq/L)	30

Considering the ongoing COVID‐19 pandemic and the presence of the highly suggestive symptoms, the patient underwent high‐resolution chest computerized tomography (HRCT) to evaluate COVID‐19 infection. The results revealed bilateral multifocal ground‐glass opacities (GGO) and mixed GGO and consolidation lesions. Because of hypoxemia and the severity of the symptoms, the patient was hospitalized. As a treatment, he received noninvasive positive pressure ventilation, hydroxychloroquine, azithromycin, and ceftriaxone. The nasopharyngeal swab test was also obtained to perform the COVID‐19 reverse transcription‐polymerase chain reaction (RT‐PCR) assay that confirmed the diagnosis.

**FIGURE 1:**
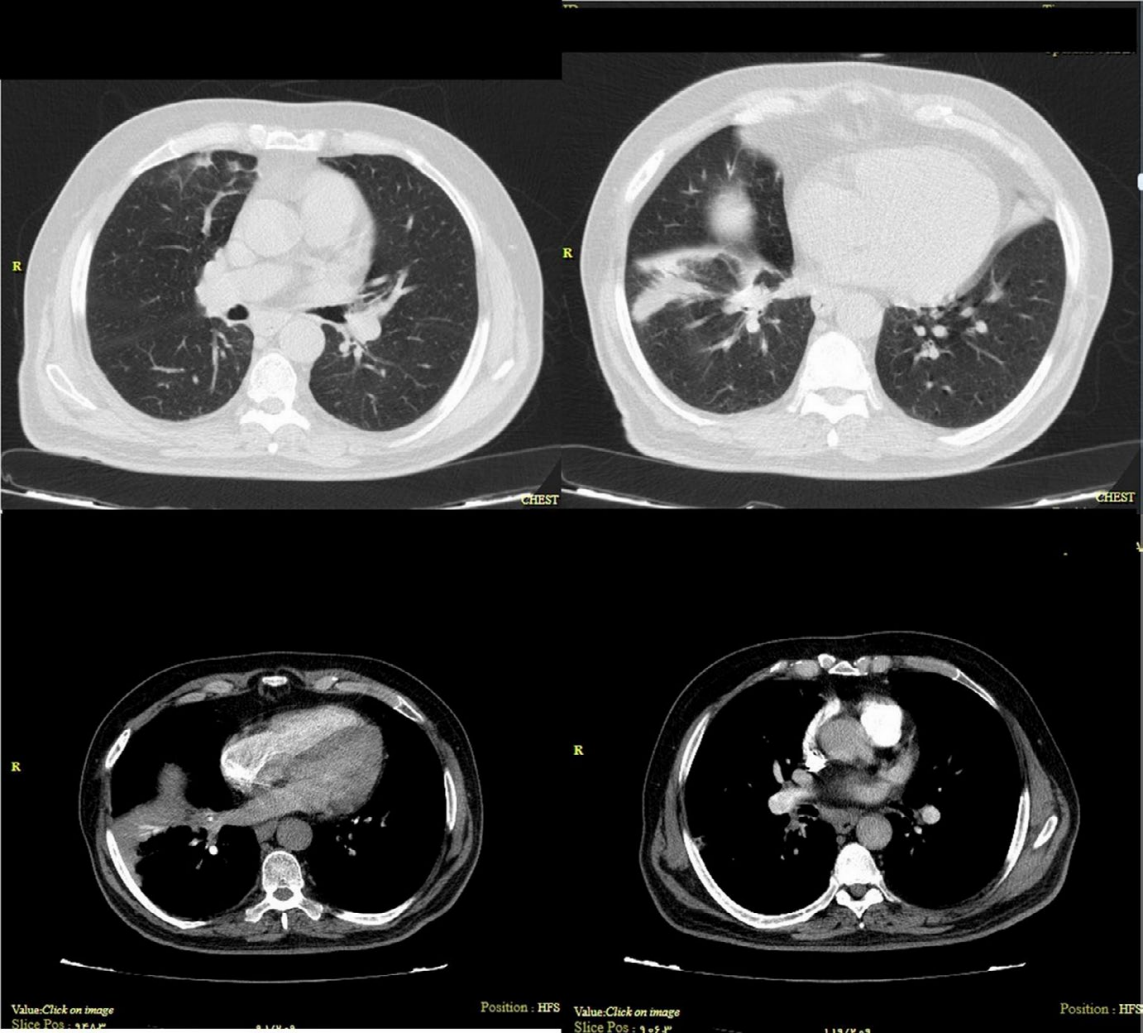
The right and left main pulmonary arteries with a massive extension into lobar arteries of both lungs

The hypoxemia persisted even after two days of hospitalization. To rule out the potential pulmonary embolism, the patient underwent CT angiography of his chest, with a consensus on safety. Results demonstrated filling defects within the pulmonary vasculature including the right and left main pulmonary arteries with a massive extension into lobar arteries of both lungs (Figure 1). Lower‐limb compression ultrasonography was negative. To assess the right ventricular function, the patient underwent echocardiography. An ejection fraction of 40% and inferior wall hypokinesia were understood. Besides, moderate enlargement of the right ventricle had resulted in a D‐shaped left ventricle (mean pulmonary artery pressure exceeded 25 mm Hg at rest).

The thrombophilia panel was requested, and since the hemodynamic status of the patient was stable, the IV heparin infusion was commenced. Also, the previous treatments continued. To rule out the presence of underlying malignancies as a predisposing factor for hyper coagulopathy state and subsequent pulmonary thromboembolism, the patient underwent a CT scan of the abdomen and pelvic. The results were normal. Also, Factor II G20210A and Factor V Leiden R506Q mutations were assessed using fluorescently labeled target‐specific probes and Light Cycler 480 instrument (Roche) in genomic DNA of the whole blood sample. Both came out negative for these mutations. Moreover, the level of anticardiolipin (IgG), anticardiolipin (IgM), protein C, protein S, antithrombin III, and D‐dimer was 7.5 U/mL (normal), 4.1 U/mL (normal), 35% (decreased), 45% (decreased), 93% (normal), and 1120 ng/ml (increased), respectively. Finally, the lupus anticoagulant was negative (Table 2).

**TABLE 2:** The patient's blood coagulation profile

Measure	Result
Protein C activity (Units/mL)	35
Protein S level (Units/mL)	45
Lupus anticoagulant	Negative
Antithrombin III activity (Units/mL)	93%
Factor II G20210 mutation	Negative
Factor V Leiden (R506Q) mutation	Negative
Anticardiolipin (IgG) (Units/mL)	7.5
Anticardiolipin (IgM) (Units/mL)	4.1
Fibrinogen level (mg/dL)	380

After one week, the fever and chills disappeared, and the oxygen saturation improved, gradually. Four weeks after the admission date, the patient was discharged with no dyspnea, while rivaroxaban outpatient was offered.

## 3 DISCUSSION

Previous studies evidenced that SARS‐CoV‐2 stimulates the coagulation pathway, resulting in abnormal coagulation parameters and endothelial dysfunction. These make the important factor of increased D‐dimer level a poor prognostic factor for patients with COVID‐19.^10^, ^11^ Also, the biopsy examination of patients who died with the diagnosis of COVID‐19 has revealed histomorphologically diffuse alveolar damage confirming the COVID‐19–induced coagulopathy.^12^

In our case, as a patient with COVID‐19 presented with pulmonary embolism without any previous predisposing hypercoagulable risk factor, the level of protein C and protein S had decreased with a considerable rise in D‐dimer. In another study by Panigada et. al.,^13^ opposite results have been reported. By assessing 24 COVID‐19 patients in the intensive care unit, they have reported an increase in the level of protein C and a marginal decrease in the level of protein S.

The kinetics and robustness of the immune response to COVID‐19 are yet to be known.^14^ Recent studies show that respiratory failure in COVID‐19 patients is not only caused by respiratory distress but also microscopic clot formation processes. This finding may be a clue to a better understanding of the treatment of these patients. There is a strong relationship between the levels of D‐dimer molecule and disease progression and CT scan findings in these patients, which indicates the cause of venous clots in them.^15^

Some studies also show that there is not a direct relationship between D‐dimer levels and disease severity. Accordingly, imaging studies have confirmed that COVID‐19 syndrome is an inflammatory, clotting‐inflammatory process that negatively affects lung function, and in later stages, affects other organs in the body.^16^

## 4 CONCLUSION

Treatment of a COVID‐19 patient caused the worsening of the clinical respiratory symptoms and persistence of hypoxemia. The high D‐dimer level due to COVID‐19 infection masks the diagnosis of pulmonary embolism infection in these patients. Thus, CT angiography of the chest should be performed to rule out the pulmonary embolism if there are no contraindications such as renal dysfunction and contrast allergy.

## 5 ETHICS APPROVAL

The ethics committee of the “Mashhad University of Medical Sciences” has approved that the current work abides by the national ethical standards for conducting medical research in Iran (Approval ID: IR.MUMS.REC.1399.499).

## CONFLICT OF INTEREST

The authors acknowledge that there is no conflict of interest.

## AUTHOR CONTRIBUTIONS

FP: performed the investigation process and data collection. ND: led the team throughout the work, written, and revised the manuscript. MM: carried out patient treatment. FA: carried out patient treatment.

## Data Availability

The data that support the findings of this study are available from the corresponding author upon reasonable request.
